# Implementation of LABSTER virtual lab in immunology for innovative teaching and improved learning in veterinary degree

**DOI:** 10.3389/fvets.2025.1603469

**Published:** 2025-08-22

**Authors:** Javier G. Casado, Raquel Tarazona, Hector Cordero

**Affiliations:** Immunology Unit, Department of Physiology, Faculty of Veterinary, Universidad de Extremadura, Cáceres, Spain

**Keywords:** LABSTER, virtual simulator, virtual lab, immunology, innovative teaching and learning, veterinary, biochemistry

## Abstract

Virtual laboratories provide a risk-free environment for students to conduct experiments, particularly those involving hazardous materials or complex procedures. Previous studies have shown that gamified elements and interactive tools enhance the interest of students and promote active participation. In the case of Immunology in the veterinary degree, our prior observations indicate that veterinary students experience learning difficulties with topics related to molecules and effector functions of the immune system. This study aimed to investigate the utility of virtual laboratory simulations for teaching Immunology. For that purpose, cohorts of second-year veterinary students and third-year biochemistry students at the University of Extremadura were surveyed before and after the implementation of the LABSTER virtual lab in the Immunology course. The survey questions addressed their perceptions of virtual environments, prior computer knowledge, experiences with virtual platforms for learning, ethics, comparisons to traditional lab practices, and the perceived utility of virtual labs for their future careers. Gender differences were considered to determine potential variations in perceptions of virtual learning. Results indicated that veterinary students had no prior experience with LABSTER or similar virtual tools in other courses. Initially, nearly half of the students felt “unprepared” to use such tools. Notably, all students rated the experience using LABSTER virtual lab with an average of 7.5 ± 1.4 and 8.1 ± 1.8, in men and women, respectively. Indeed, most students perceived that LABSTER was useful for learning Immunology. Most students were hesitant about fully replacing traditional labs with virtual tools but found LABSTER beneficial for their future career skills. In conclusion, while virtual simulations are not a complete substitute for hands-on laboratory experiences, they may effectively help students develop practical skills and familiarize themselves with laboratory procedures and equipment. These findings highlight the potential of virtual labs as a complementary tool in veterinary education.

## 1 Introduction

Immunology is a fundamental discipline in veterinary education, being essential for understanding the body’s defense mechanisms against infectious diseases, and its prevention in animals. The teaching of immunology in veterinary degrees has significantly evolved in recent decades, incorporating new pedagogical methodologies and educational technologies. In the case of veterinary immunology curricula, this subject comprises a wide range of topics, going from basic fundaments of innate and adaptive immunity to clinical applications in immunotherapy.

Although the structuring and emphasis of certain immunology contents may vary between institutions (1), the integration of virtual tools in immunology teaching has gained significant attention in recent years, particularly with the rise of on-line and hybrid learning models. Previous studies have evaluated the student preferences between face-to-face and on-line laboratory sessions, for optimizing learning experiences in immunology courses (2). In 2013, the group of Alves et al. developed virtual on line laboratories to learn basic concepts of immunology (3). More recently, these researchers have developed a software to enhance the learning of antigen–antibody interactions (4). Other groups have been focused in e-learning platforms and virtual labs, offering interactive environments for exploring human-artificial immune systems, providing students with immersive and accessible learning experiences (5). These developments underscore the growing role of virtual technologies in complementing traditional teaching for immunology education.

Addressing gender bias in STEM (Science, Technology, Engineering, and Mathematics) and digital education is increasingly recognized as a critical component of educational research and practice. Persistent gender disparities in access to, engagement with, and representation within STEM fields continue to shape educational outcomes and professional trajectories. These disparities are further compounded by the digital gender divide, which may affect students’ self-perception of technological competencies and their willingness to engage with virtual learning environments (6, 7). Integrating a gender perspective into educational studies is therefore essential to promote inclusive, equitable, and evidence-based teaching practices.

Apart from immunology, other scientific areas have used augmented reality, mixed reality or virtual reality with beneficial effects for medical students (8). Interestingly, during the COVID-19 pandemia, these digital platforms offered to students the opportunity to engage in realistic laboratory experiences without the constraints of physical space, time, or resources (9). Recent studies have also demonstrated the efficacy of virtual labs in enhancing student learning outcomes, motivation, and practical skills across various scientific disciplines (10–13). More specifically, virtual labs have been particularly valuable in microbiology (14, 15), biotechnology (16), and pharmaceutical sciences (17, 18), allowing students to visualize complex molecular processes.

In the case of Immunology for veterinary students, virtual lab simulations could be an interesting option and should be explored to improve learning outcome, motivation and practical skills. In this study, we firstly aimed to evaluate the learning difficulties of veterinary students in Immunology subject. Multiple-choice questions were categorized into different topics revealing learning difficulties in “Molecules of the immune system” and “Function/mechanisms of the immune system.” Based on that, we introduced the virtual simulator LABSTER for veterinary and biochemistry students to acquire knowledge in immunoassays, immune cells interactions and immune responses among others. These students completed a survey about their perception of the utility and impact of LABSTER virtual lab in learning immunology using a customized questionnaire with questions about ethics, comparison with traditional lab practices and other science-specific questions, and assuming the limitations in absence of validated questionnaires on this matter.

In summary, our results revealed that virtual simulations using LABSTER seem to be useful not only to improve the confidence of veterinary and biochemistry students with technology but also for learning certain concepts of immunology and laboratory techniques. Although most students were open to exploring LABSTER in other subjects, up to 70% of them disagreed in fully replacing physical practices with virtual ones. On a positive note, the great majority of students considered LABSTER “useful” or “very useful” for their future career. Overall, our study revealed that students enhanced their learning experience in immunology and complemented the skills gained through traditional practices.

## 2 Materials and methods

### 2.1 Subjects

A cohort of 90 s-year veterinary students (20.9 ± 0.2 years) and a separate cohort of 32 third-year biochemistry students (20.9 ± 2.6 years), both at the Faculty of Veterinary (University of Extremadura, Spain) were surveyed about LABSTER virtual lab in the Immunology course.

### 2.2 Virtual lab

We purchased a total of 15 licenses of LABSTER ApS (Denmark) installed in local computers at the Faculty of Veterinary. The practice was divided into three virtual simulations: “Organs and cells of the immune system,” “Immunoassay to detect SARS-CoV-2 antibodies” and “Antibodies: Why are some blood types incompatible?,” matching some of the areas where students used to fail in Immunology course in previous years.

### 2.3 Surveys

Students were anonymously surveyed using the platform of Microsoft Forms before and after the implementation of the LABSTER virtual lab in the Immunology course within their academic programs. The surveys were designed by the collaboration of three immunology educators framing the survey questions into four different categories: perceived learning and digital readiness, ethics, future and personal data. The survey questions addressed their perceptions of virtual environments, prior computer knowledge, experiences with virtual platforms for learning immunology and other subjects, ethics, comparisons to traditional lab practices, and the perceived utility of virtual labs for their future careers. Age and sex of the students were also recorded in the questionnaires. The survey details (types of questions, response scales, and the rationale for its design) are included in Supplementary Table 1. It is important to note that this self-designed survey does not adhere to established psychometric validation standards. Moreover, our survey includes items with diverse formats, scales and scores, so Cronbach’s alpha could not be performed.

### 2.4 Gender-based analysis

The answers of veterinary students were also analyzed separately by men (*n* = 26) and women (*n* = 64) to determine potential variations in perceptions of virtual learning. Due to the lack of men in biochemistry degree, they were excluded from the gender-based analysis.

### 2.5 Statistical analysis

Results of the numerical variables were expressed as mean ± SD and as percentages (%). Results of the categorical variables were expressed as percentages (%). All the statistical analyses were performed using GraphPad Prism software version 8.0. Shapiro–Wilk normality test was conducted to determine the appropriate test: parametric if *p* > 0.05 (indicating normality) or non-parametric if *p* < 0.05 (indicating non-normality). Subsequently, Student’s t-test for parametric data or the Mann–Whitney U test for non-parametric data were selected, as applicable. In addition, Cohen’s D effect size and post-hoc power were calculated for our datasets.

## 3 Results

### 3.1 The use of LABSTER improved the self-perception of information technology knowledge

The implementation of LABSTER virtual lab requires the use of an individual computer, access to internet, and a basic to intermediate level of information technology (IT) knowledge. Notably, none of the surveyed students had prior knowledge and never experienced any practice with LABSTER virtual lab in their career. We next asked the students to rate on a scale from 0 to 10 their self-perception of IT skills before and after using LABSTER virtual lab.

**Figure 1:**
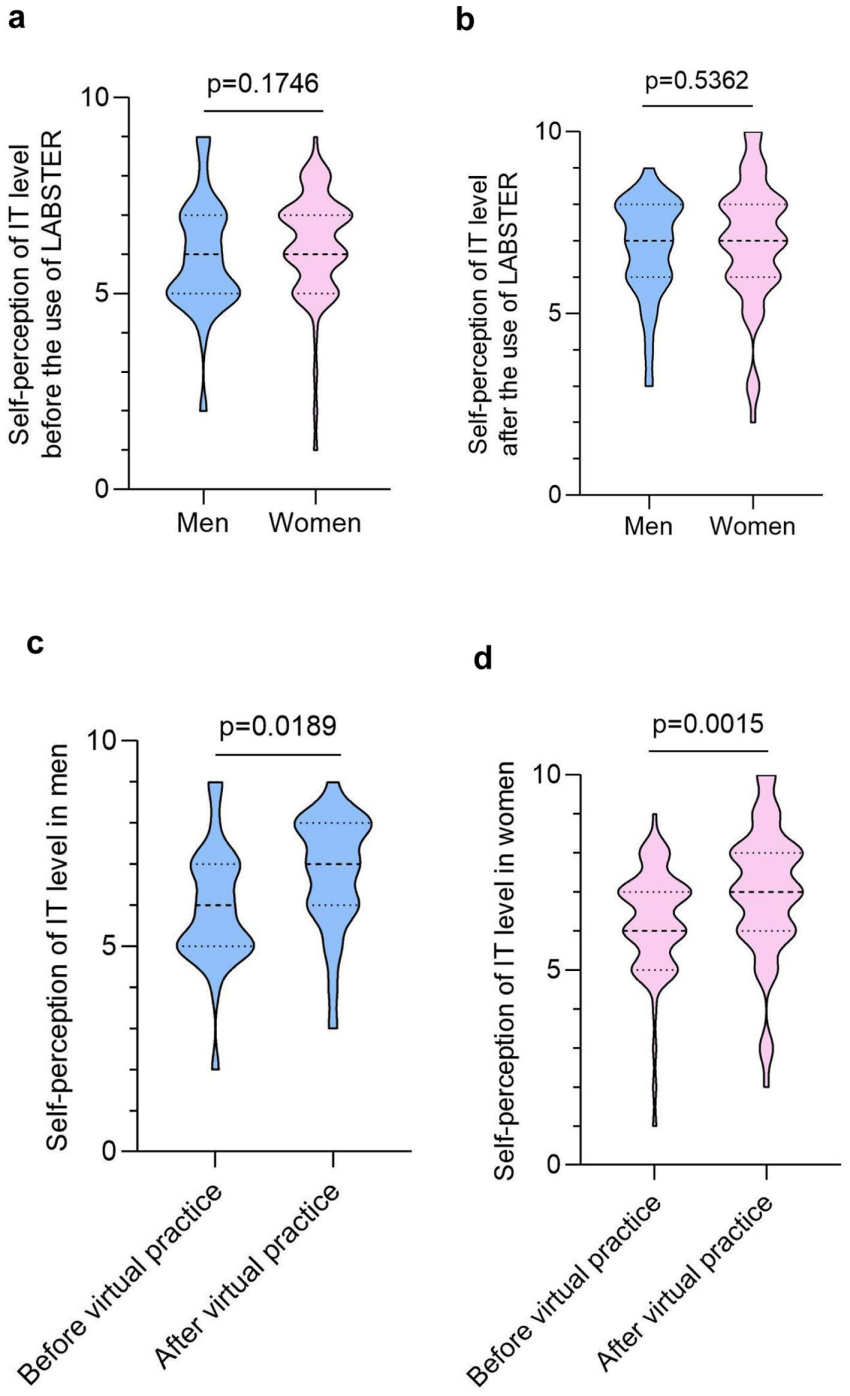
The use of LABSTER improved self-perception of IT knowledge in veterinary. Violin plots showing the self-perceived IT knowledge of veterinary students before **(a)** and after **(b)** using LABSTER, separated by gender (*n* = 90, 26 males and 64 females). Comparison of pre- and post-LABSTER self-perception IT knowledge scores in male **(c)** and female **(d)** students (*n* = 90, 26 males and 64 females). Normality was tested using Shapiro–Wilk test. Differences were considered statistically significant when *p* < 0.05 using the non-parametric Mann–Whitney U test. The effect size was −0.2 **(a)**, −0.14 **(b)**, −0.58 **(c)** and −0.52 **(d)**. Post-hoc power was 0.3% **(a)**, 9.3% **(b)**, 54.7% **(c)** and 83.9% **(d)**.

Before using the LABSTER, both male and female students had similar self-perception of IT knowledge, with averages of 5.9 ± 1.5 and 6.2 ± 1.4, respectively (Figure 1a). After the practice, the veterinary students exhibited increased confidence in their technological abilities, with average scores rising to 6.8 ± 1.4 in men and 7.0 ± 1.7 in women (Figure 1b). A significant improvement was observed when comparing pre- and post-LABSTER self-ratings for both male (Figure 1c) and female students (Figure 1d).

### 3.2 The implementation of LABSTER was promising for learning immunology

**Figure 2:**
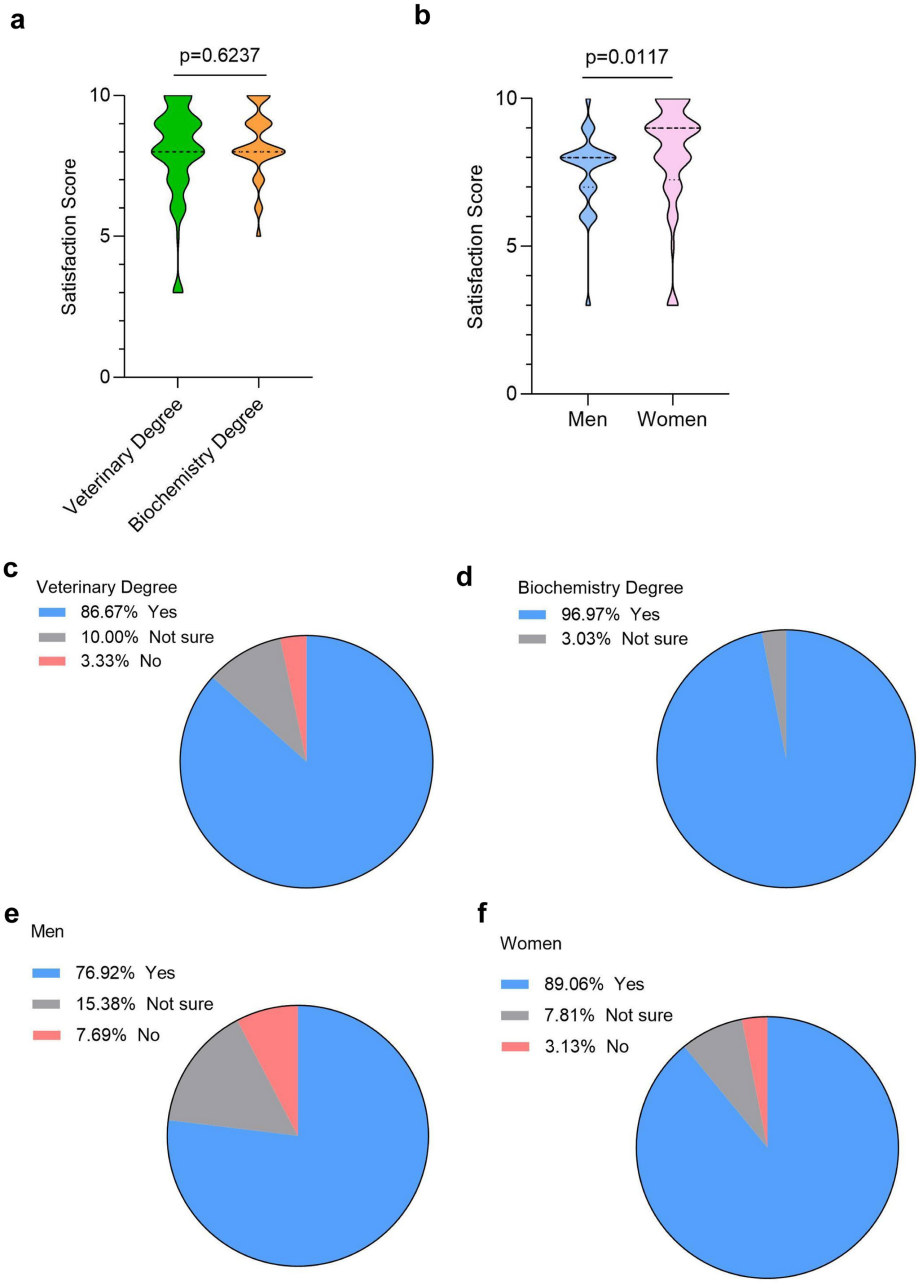
Student satisfaction with LABSTER and its effectiveness for learning immunology. **(a)** Overall satisfaction scores from veterinary and biochemistry students (*n* = 122, 90 vet students and 32 biochemistry students) **(b)** Satisfaction scores in veterinary students by gender (*n* = 90, 26 males and 64 females). Percentage of veterinary **(c)** and biochemistry **(d)** students found LABSTER useful for learning immunology (*n* = 122, 90 vet students and 32 biochemistry students). Percentage of male **(e)** and female **(f)** veterinary students who considered LABSTER useful (*n* = 90, 26 males and 64 females). Normality was tested using Shapiro–Wilk test. Differences were considered statistically significant when p < 0.05 using the non-parametric Mann–Whitney U test. The effect size was −0.19 **(a)** and 0.36 **(b)**. Post-hoc power was 15.6% **(a)** and 33.6% **(b)**.

The success of LABSTER’s implementation in the Immunology course was evaluated based on student satisfaction ratings. Students from both veterinary and biochemistry degrees were asked to rate their satisfaction with LABSTER on a scale from 1 to 10. Importantly, the results show a great satisfaction score with an average of 8.0 ± 1.7 in veterinary students and 8.3 ± 1.2 in biochemistry students. It is interesting to note that no student of biochemistry degree rated the level of satisfaction below 5 while four veterinary students rated the level of satisfaction in 3 of a total of 10 (Figure 2a).

Regarding gender differences, female students rated the LABSTER experience slightly higher than their male counterparts, with average scores of 8.1 ± 1.8 and 7.5 ± 1.4, respectively. However, the number of dissatisfied students was minimal in both sexes (Figure 2b).

To know how effective the implementation of LABSTER virtual lab was for learning Immunology, we asked the students after the practice if the virtual program was useful for learning key concepts of the subject. Among veterinary students, 86.7% agreed that LABSTER was beneficial for learning immunology (Figure 2c), while in the biochemistry cohort, this percentage was even higher at approximately 97% (Figure 2d). Gender-based differences were also observed, with 77% of male veterinary students agreeing on its usefulness compared to 89% of female students (Figures 2e,f).

### 3.3 Reduction of the number of animals in biomedical practices

**Figure 3:**
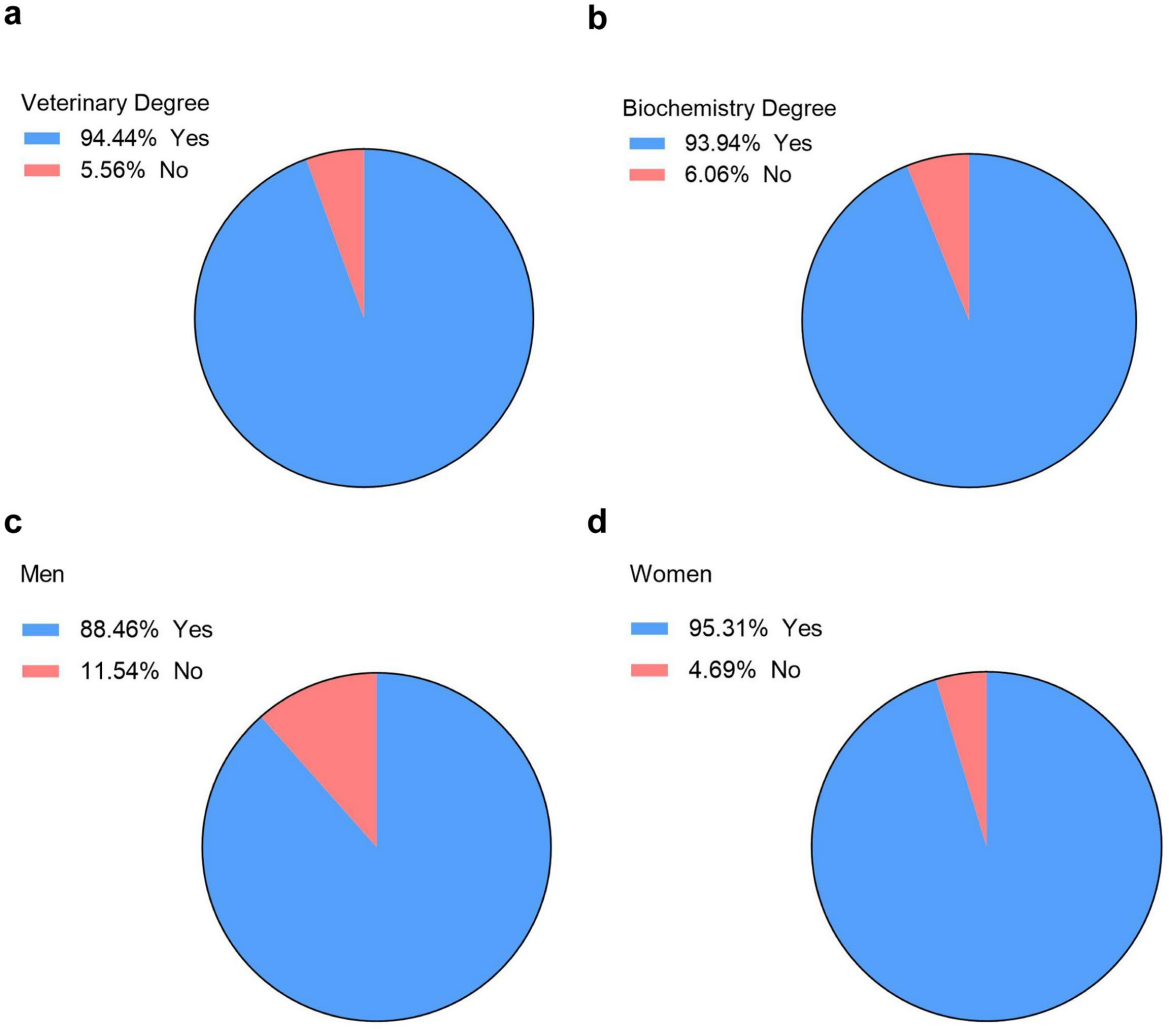
The role of virtual labs such as LABSTER in reducing the number of animals used in biosciences. Percentage of veterinary **(a)** and biochemistry **(b)** students who believe LABSTER helps minimize animal use (*n* = 122, 90 vet students and 32 biochemistry students). Percentage of male **(c)** and female **(d)** students who believe LABSTER helps minimize animal use (*n* = 90, 26 males and 64 females).

The terms replacement, reduction, and refinement (3 Rs) for minimizing the potential for animal pain and distress in biomedical research was coined for the first time in 1959 by Russell and Burch in the article “The Principles of Humane Experimental Technique.” Since then, the 3 Rs principle has become a cornerstone of regulations and ethical protocols involving animals. To explore the potential role of virtual labs in reducing animal use, students were asked whether LABSTER could contribute to this objective. The majority agreed, with 94.4% of veterinary students (Figure 3a) and 93.9% of biochemistry students (Figure 3b) affirming the benefits of virtual labs in minimizing animal use.

When analyzed by gender, 88.5% of male students (Figure 3c) and 95.3% of female students (Figure 3d) supported the idea that virtual labs could effectively reduce the number of animals used in experimentation and teaching.

### 3.4 Explore LABSTER in other subjects of the academic program

**Figure 4:**
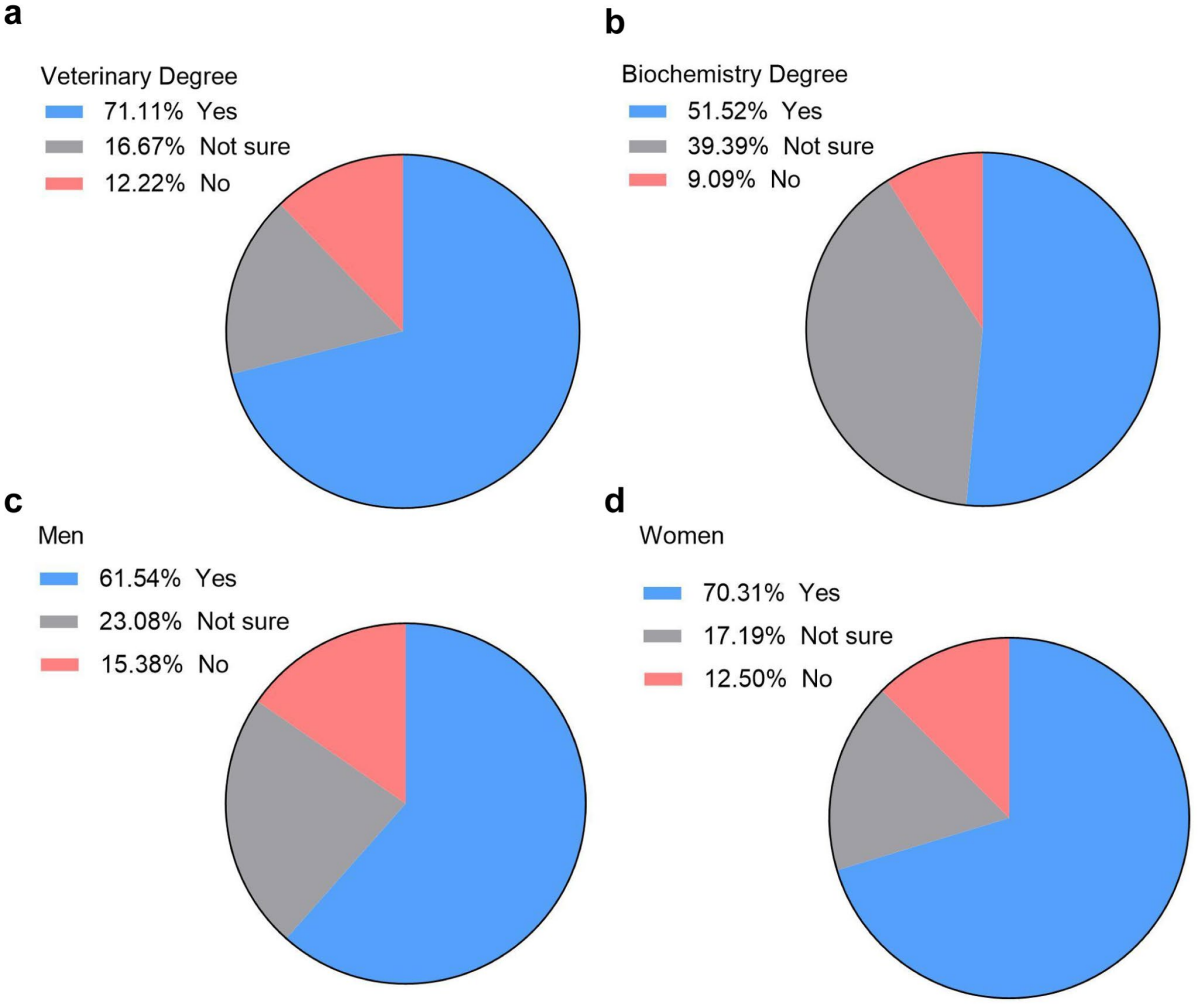
Interest in expanding LABSTER virtual lab to other subjects in veterinary sciences. Percentage of veterinary **(a)** and biochemistry **(b)** students interested in using LABSTER in other subjects (*n* = 122, 90 vet students and 32 biochemistry students). Interest in LABSTER expansion among male **(c)** and female **(d)** students (*n* = 90, 26 males and 64 females).

As LABSTER was introduced for the first time in the Faculty of Veterinary Science for Veterinary and Biochemistry Degrees, students were asked whether they would like to use LABSTER in other subjects. Veterinary students were enthusiastic about the idea, with 71% of them answering yes, the rest of them were equally split between not sure and no (Figure 4a). Less enthusiasm was shown in biochemistry students where only 51.5% were positive about the idea of exploring a virtual lab in other subjects (Figure 4b).

Gender analysis revealed that both male and female veterinary students were generally interested in extending LABSTER to other subjects, though the percentage was higher among female students (70.3%) compared to male students (61.5%) (Figures 4c,d).

### 3.5 Is a virtual practice with LABSTER more useful than a hands-on lab practice?

**Figure 5:**
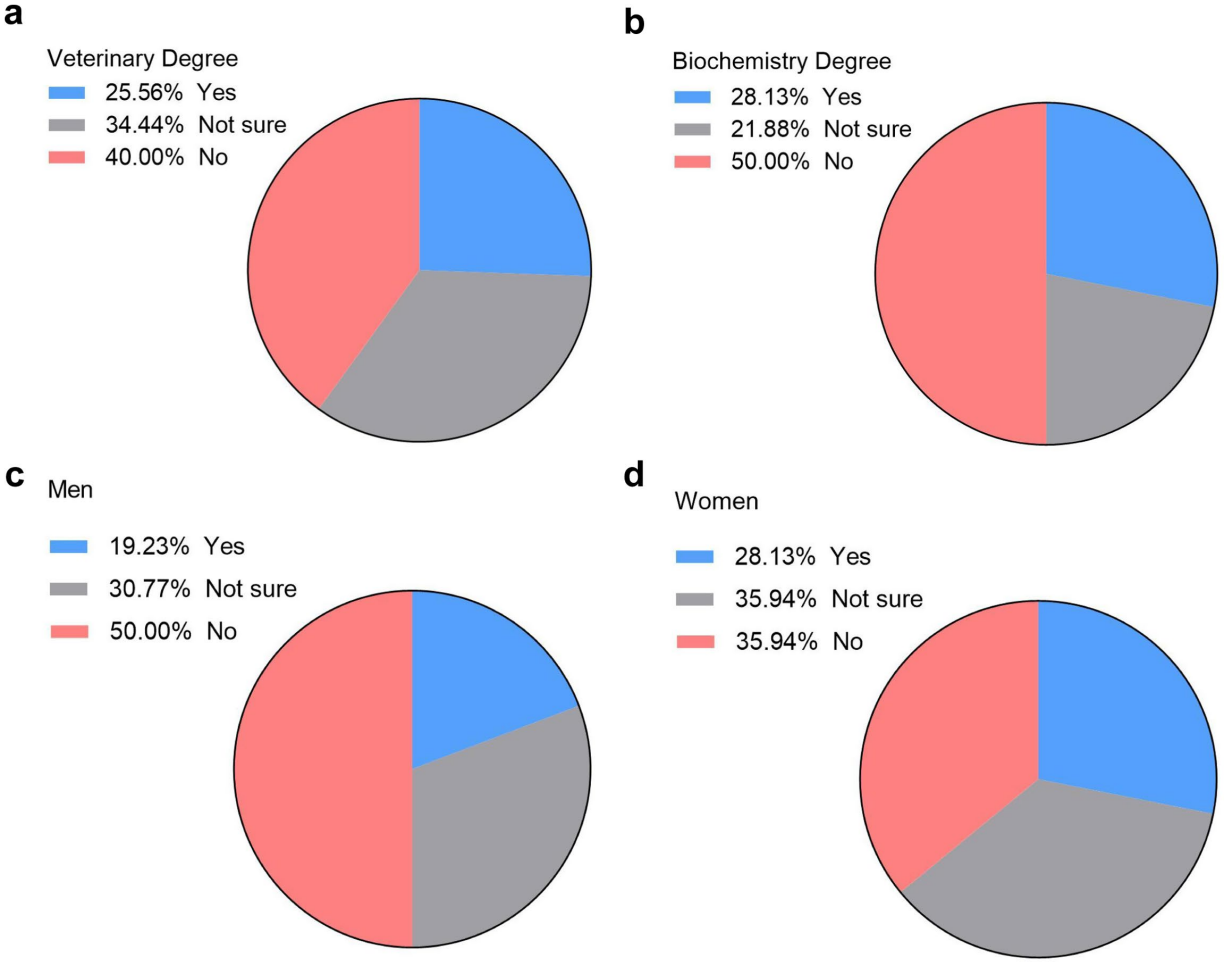
Student perspectives on virtual versus hands-on lab practices. Opinions of veterinary **(a)** and biochemistry **(b)** students on whether LABSTER is more useful than hands-on lab practice (*n* = 122, 90 vet students and 32 biochemistry students). Percentage of male **(c)** and female veterinary students on whether LABSTER is more useful than hands-on lab practice (*n* = 90, 26 males and 64 females).

With digitalization being a key objective of European regulations, we examined the extent to which virtual learning tools can replace traditional hands-on practices. We asked the veterinary and biochemistry students if virtual learning tools like LABSTER can be more useful than hands-on lab practice. Opinions were quite divided in veterinary students, with 25.6% of them agreeing that LABSTER can be more useful than hands-on lab practice, 34% of them were hesitant to it, and a significant 40% were against this idea (Figure 5a). More striking were the answers of biochemistry students with half of them thinking that hands-on lab practice is more useful than virtual lab practices (Figure 5b).

Gender differences emerged within the veterinary cohort about if virtual lab practices are better than hands-on lab practices. While only 19% of men students supported this idea and 50% were against it (Figure 5c), 28% of women students seems to agree with the fact that virtual labs can be better than hands-on labs, and 36% disagreed (Figure 5d).

### 3.6 Replacing traditional lab practices: a controversial issue

**Figure 6:**
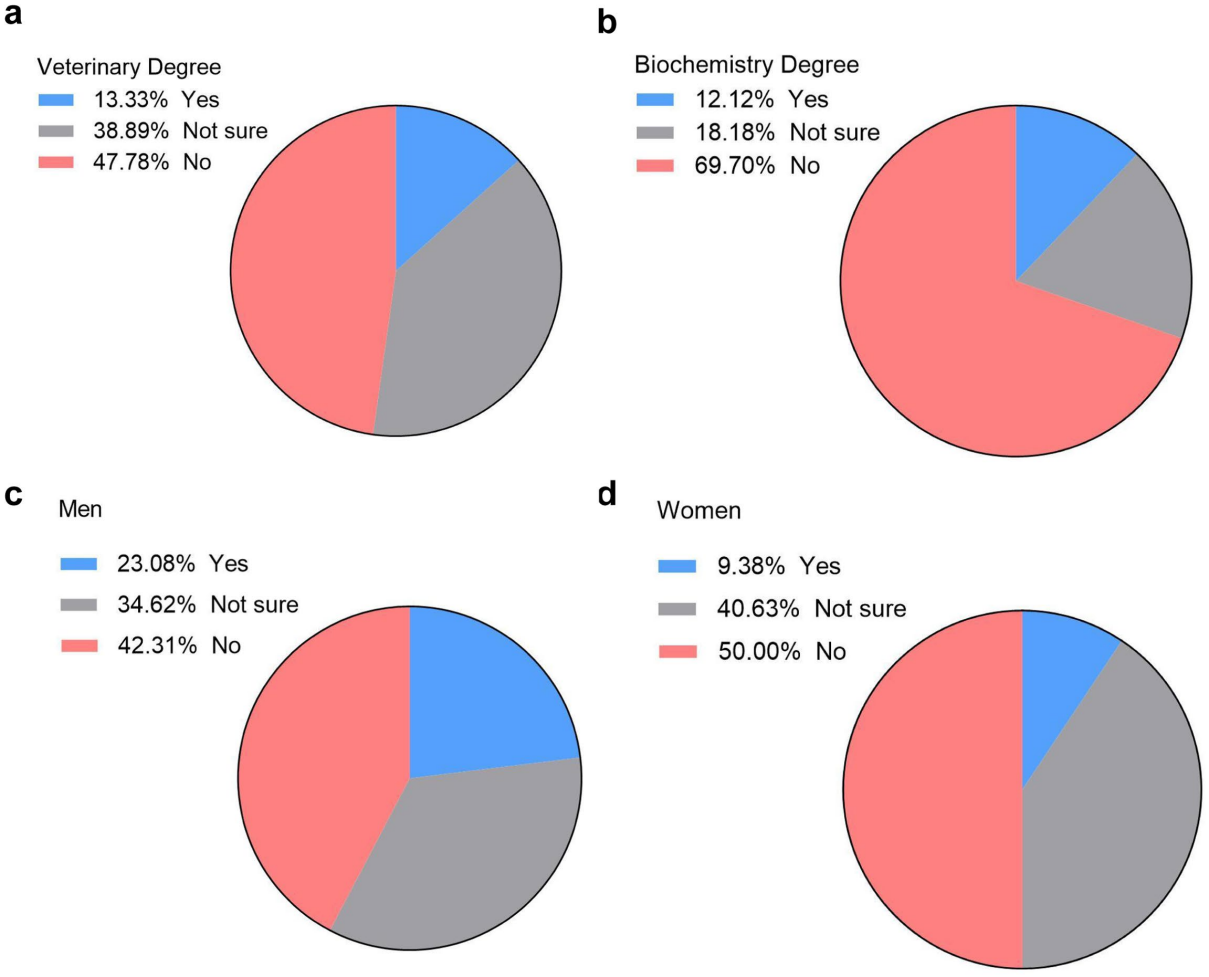
Student perspectives on replacing hands-on lab practices with virtual labs. Preferences expressed as percentages of veterinary **(a)** and biochemistry **(b)** students regarding replacing hands-on lab practices with virtual ones (*n* = 122, 90 vet students and 32 biochemistry students). Preferences expressed as percentages of male **(c)** and female **(d)** veterinary students on replacing hands-on lab practices (*n* = 90, 26 males and 64 females).

The integration of digital tools in education raises ethical and pedagogical concerns. To explore student perspectives, we asked veterinary and biochemistry cohorts if they prefer to replace lab practices with virtual learning tools like LABSTER in other subjects. The results were overwhelming, with 47.8% of veterinary students opposed to the idea of replacing hands-on lab practices and 39% of them were not sure if they would like this idea or not (Figure 6a). On the other hand, biochemistry students were even more concerned about the idea of replacing hands-on lab practices by virtual lab practices, with 70% of them replied no while 18% were not sure (Figure 6b).

Gender-based analysis revealed that male students were more open to replacing hands-on lab practices, with 23% in favor (Figure 6c). In contrast, only 9% of female students supported this transition (Figure 6d). Additionally, half of female students opposed the idea, compared to 42% of male students.

### 3.7 Virtual labs help to acquire skills for the future of veterinary students

**Figure 7:**
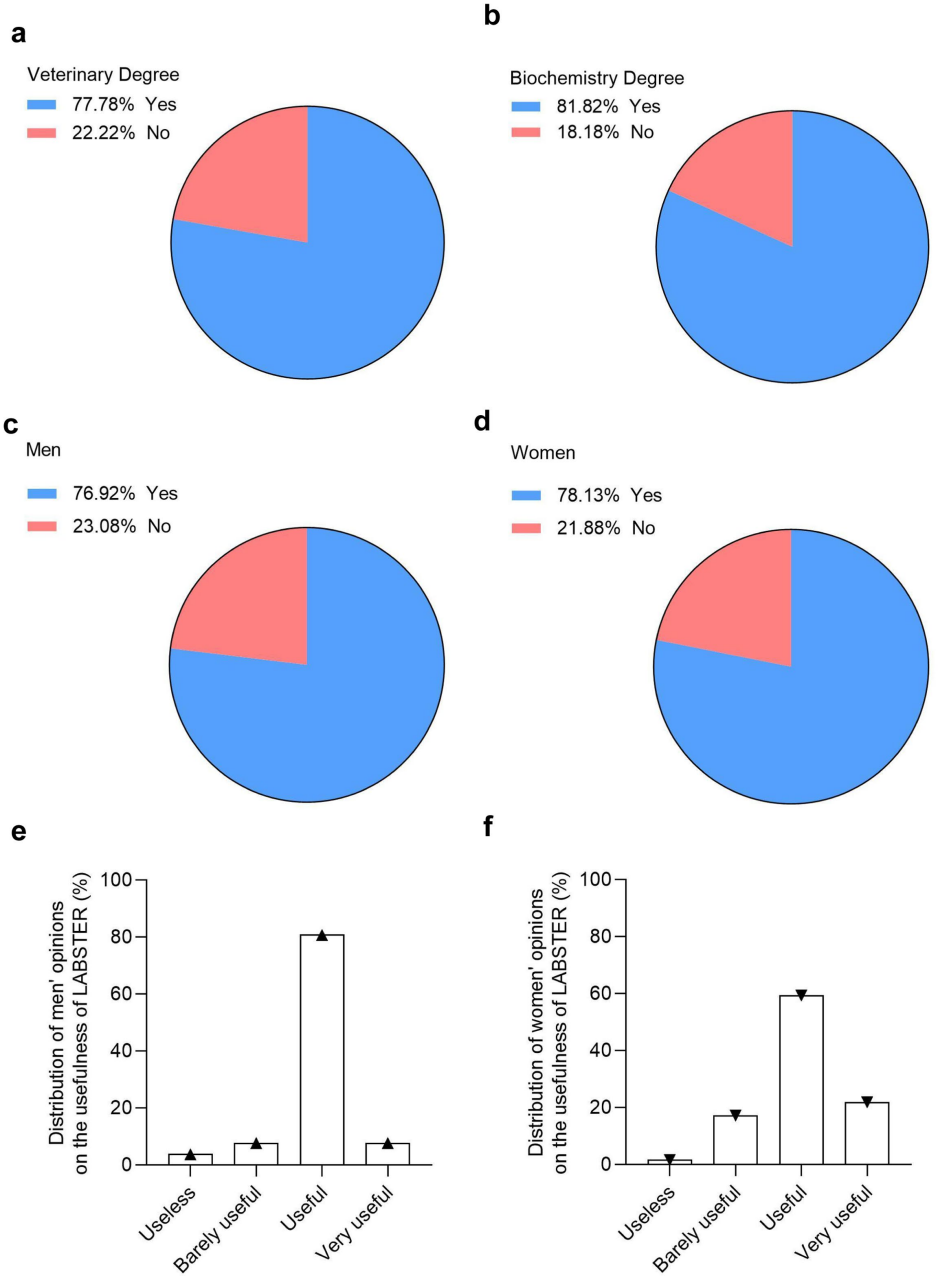
Student perspectives on how LABSTER virtual lab help them develop the practical skills needed for their future profession. Percentage of veterinary **(a)** and biochemistry **(b)** students who believe LABSTER enhances their practical skills for the future (*n* = 122, 90 vet students and 32 biochemistry students). Percentage of male **(c)** and female **(d)** students who believe LABSTER enhances their practical skills for the future (*n* = 90, 26 males and 64 females). Distribution in opinions in male **(e)** and female **(f)** veterinary students about how useful LABSTER is for their future careers (*n* = 90, 26 males and 64 females).

The ultimate goal of working on innovative teaching methods is to develop skills and competences useful for the future career of the students. In this regard, we asked the veterinary and biochemistry students whether the LABSTER virtual lab will help them develop the practical skills needed for their future profession. The results from our survey showed that 77.8% of veterinary students perceived that the use of LABSTER helps to develop practical skills needed for the future profession (Figure 7a). Similarly, 81.8% of biochemistry students also agreed that the use of LABSTER helps for developing skills in their future jobs (Figure 7b).

Unlike previous topics, gender differences were minimal in this case, with 77% of male veterinary students and 78% of female students agreeing that LABSTER enhanced their future competencies (Figures 7c,d). Finally, when veterinary students were asked to rate the usefulness of LABSTER for their careers, more than 80% of male students considered it beneficial (Figure 7e). Among female students, responses were more varied with 22% rated it as very useful, 60% as useful, and 17% as barely useful (Figure 7f).

## 4 Discussion

This study aimed to investigate the utility of virtual laboratory simulations for teaching Immunology in veterinary students. We have introduced the ‘LABSTER virtual lab’ to a large group of students with a veterinary degree and biochemistry degree. To evaluate certain aspects such as satisfaction score, acquisition of laboratory skills or the utility of this technology for the reduction of experimental animals, the students completed a survey before and after using this interactive tool. Additionally, this survey aimed to evaluate the students’ opinion for the hypothetical applicability of LABSTER for other subjects and to determine whether it could effectively be useful to replace (at least in part) conventional laboratory practices. Although validated questionnaires (i.e., “Technology acceptance model” or “Student Perceptions of Learning Environments”) are valuable instruments to ensure reliability and validity, we considered that our tailor survey was more appropriate to evaluate aspects related with ethics, comparisons to traditional lab practices, and their utility for their future careers.

Artificial intelligence and virtual laboratories enhance accessibility and active learning in science education but face challenges in integration and cost. They effectively complement hands-on labs but should be carefully introduced to ensure a proper integration (19, 20). In the case of LABSTER, it has been widely used in different disciplines such as microbiology (9, 15) or pharmacology (21) and in different University degrees such as BSc Bioscience, BSc Pharmaceutical Science and BSc Biopharmaceutical Science (22). LABSTER has been also introduced for laboratory technicians (23). However, to the best of our knowledge, this is the first study evaluating LABSTER in the field of Immunology for veterinary students, with a focus on gender-based differences. Additionally, we compared the experience of using LABSTER with a group of students from biochemistry degree. These comparisons had an exploratory nature and limited generalizability based on the low-powered results.

Our first set of results were focused on their self-perception of Information Technology level (IT). The survey was completed just before and after using virtual lab. Before using LABSTER, any difference was observed when compared men and women, surprisingly, men and women revealed a significant increase of their self-perception of IT level after virtual practice. Previous studies found in bibliography did not collect information on participants’ gender (10, 15) and some of them were uniquely focused in female students (12). In the case of the paper from Wismer et al. (24) they considered the gender dimension in the evaluation of LABSTER for the assessment of laboratory skills, and coincident with our results, their analysis did not revealed any significant difference when compared men and women.

In our study we also aimed to determine the satisfaction score of using this education tool for learning Immunology. Our results showed that veterinary students and biochemistry students reported very high satisfaction scores. A vast majority of students reported LABSTER very useful for learning Immunology. In line with our observations, previous experiences using immersive laboratory simulations have also showed very high motivation and engagement among students (10). Other studies have also demonstrated that virtual laboratories facilitated their individualized learning in health sciences laboratories (25). The gender-based analysis revealed that a higher percentage of women considered that LABSTER very useful.

Once evaluated the satisfaction score and the utility of LABSTER for learning Immunology, our survey aimed to explore the students´ perception of this tool to reduce the use of experimental animals in education. It is well known that the 3Rs framework (Replacement, Reduction, and Refinement) is necessary to promote more ethical approaches in teaching and animal research (26). As expected, our results demonstrated that veterinary and biochemistry students believed that virtual tools could reduce the number of experimental animals in their practices, and no significant differences were observed between men and women. Previous studies using computer-based virtual laboratory simulations have also supported these tools for the ethical use of animals (11). Other authors have also demonstrated that students prefer hands-on animal use, but virtual activities are still effective for learning physiology (27). An interesting analysis of Lemos et al. showed that participants, despite limited virtual reality experience, appreciated its potential to enhance procedures and experimental demonstrations. Moreover, those students with laboratory animal science experience were particularly convinced of virtual reality’s value in supporting the 3Rs principle and educational purposes (28).

The survey also aimed to evaluate if LABSTER could be introduced in other disciplines or subjects. In this case, we found significant differences when compared students from veterinary degree with students from biochemistry degree. About half of biochemistry students believed that LABSTER could be useful for other subjects, whereas a higher percentage of veterinary students shared this opinion (no differences were observed when compared males and females). Similarly, 69% of biochemistry students considered that LABSTER practices should not replace conventional laboratory practices, and this percentage was significantly lower in veterinary students. Although there are many studies demonstrating that virtual labs could be helpful for hands-on sessions and wanted to use it again (29, 30), only a few studies have compared this aspect in students from different degrees or university programs.

Our interest was focused on the utility of LABSTER for the acquisition of skills for their professional future. This question revealed similar answers when compared biochemistry with veterinary students and when compared men with women. Basically, the majority of students reported that laboratory simulations provide better technical skills for their future. These results are in agreement with a previous study in which the virtual laboratory simulations may be considered an effective tool for developing non-cognitive and cognitive skills, especially in scientific writing skills (12).

Finally, it is interesting to cite and describe a systematic review focused on the question “Are virtual physiology laboratories effective for student learning?” (31) and a meta-analysis which evaluated the “Effectiveness of virtual laboratory in engineering education” (13). The systematic review from Zhang et al. and the meta-analysis from Li and Liang included 13 and 22 peer-reviewed articles, respectively, and their conclusions were closely coincident with our results. Firstly, the utility of virtual laboratories should be evaluated in terms of learning achievement and research skills. Secondly, future studies should employ rigorous methodologies, large samples, quantitative assessments, and rigorous data analysis among others. Third, although virtual laboratories such as LABSTER could be effective for conceptual learning, their impact on motivational and technical learning remains inconclusive. In this context, one limitation we identified is the absence of a simultaneous control group, as all students must be granted access to the virtual laboratory. However, two surveys were conducted, one before and one after the virtual laboratory experience, which may serve as an alternative to using a simultaneous control group and help ensure consistency in repeated key responses. This approach was chosen due to university regulations and to promote equity in opportunities and outcomes. Our future studies in veterinary and biochemistry students will be focused on the establishment of correlations between the results of virtual laboratory activities and objective learning assessments of the immune system.

## 5 Conclusion

According to the students, the implementation of LABSTER virtual lab was perceived as positive in the context of learning immunology from a safety and virtual perspective, useful for reducing animals in veterinary sciences and for acquiring skills and confidence in using technology for their future career goals. In general, females rated better the experience with the virtual lab pointing to differences in gender perception. The full potential of virtual labs needs to be further evaluated, with improved and more realistic experiences in different areas of veterinary and biomedical sciences. Future studies for the implementation of virtual tools in the context of learning immunology will be performed using validated questionaries and incorporating objective evaluations of learning outcomes.

## Data Availability

The raw data supporting the conclusions of this article will be made available by the authors, without undue reservation.
