# Supplementary material for: Implementation of LABSTER virtual lab in immunology for innovative teaching and improved learning in veterinary degree
Source: Front Vet Sci. 2025 Aug 22;12:1603469. doi: 10.3389/fvets.2025.1603469 (PMC12411196; doi:10.3389/fvets.2025.1603469)
Supplement: Supplementary file 1 [file Table_1.docx]

**Supplementary Table 1.** Survey questions about students' perceptions of the utility and impact of the LABSTER virtual lab in learning immunology and developing laboratory skills.

| **Survey item** | **Question** | **Answers** |
| --- | --- | --- |
| Question 1 | Have you ever used other virtual applications to learn other subjects? | Yes / No / Not sure |
| Question 2 | How prepared are you to explore and learn how to use LABSTER? | Highly prepared / Prepared / Somewhat prepared / Not prepared at all |
| Question 3 | Select your computer proficiency level on a scale of 1 to 10 | 1 to 10 |
| Question 4 | Do you think that virtual tools such as LABSTER can be useful for learning this subject? | Yes / No / Not sure |
| Question 5 | Do you think virtual learning tools like LABSTER can be more useful than a practice in the lab? | Yes / No / Not sure |
| Question 6 | Would you prefer other subjects to replace laboratory practices with virtual learning tools such as LABSTER? | Yes / No / Not sure |
| Question 7 | Do you think virtual learning tools like LABSTER will help you develop practical skills needed for your future career? | Yes / No |
| Question 8 | Select the level of usefulness of virtual learning tools like LABSTER for your future work | Highly useful / Useful / Somewhat useful / Not useful at all |
| Question 9 | Do you think that the use of virtual learning tools such as LABSTER can have a benefit in reducing the use of animals in experimentation and teaching? | Yes / No |
| Question 10 | Evaluate your level of satisfaction with the LABSTER practical activity in the Immunology course. | 1 to 10 |
| Question 11 | Select your current age | 0 - 99 |
| Question 12 | What is your gender? | Male / Female / NA |
| Question 13 | Select your degree | Veterinary / Biochemistry |

NA: No Answer
